# Calibration Method for Large-Aperture Antenna Surface Measurement Based on Spatial Ranging Correction

**DOI:** 10.3390/s26010312

**Published:** 2026-01-03

**Authors:** Xuesong Chen, Yaopu Zou, Changpei Han, Xiaosa Chen, Linyang Xue, Fei Wang

**Affiliations:** 1 University of Chinese Academy of Sciences, Beijing 100049, China; chenxuesong231@mails.ucas.ac.cn (X.C.);; 2 Shanghai Institute of Technical Physics, Chinese Academy of Sciences, Shanghai 200083, China; zyp@mail.sitp.ac.cn (Y.Z.);; 3 State Key Laboratory of Infrared Physics, Shanghai Institute of Technical Physics, Chinese Academy of Sciences, 500 Yutian Road, Shanghai 200083, China

**Keywords:** geometric structural error, nonlinear optimisation, optical axis deviation, spatial ranging correction

## Abstract

To address the accuracy calibration issue of the high-precision FMCW laser scanning measurement system for the large-aperture antenna of the Fengyun-4 microwave sounding satellite in orbit, this paper proposes a system calibration method based on space ranging correction. First, by analyzing the geometric structure and optical axis offset errors of the FMCW measurement system, a comprehensive error model comprising 13 key parameters was established. Second, a calibration field was constructed using a high-precision reference scale and planar targets. The spatial ranging correction method was employed to eliminate reliance on the accuracy of reference point coordinates inherent in traditional approaches, and nonlinear least-squares optimization was used to estimate the error parameters. Finally, a calibration scheme involving four operational conditions was implemented, with validation performed under three independent operational conditions. Experimental results show that the RMS error in relative distance between two points decreased from 17.5 mm to 2.3 mm after calibration. The ICP registration residual for the spatial point cloud was reduced to 2.5 mm, and point cloud shape fidelity improved by 86.6%. This validates the effectiveness and generalization capability of the proposed method. This research provides a reliable technical approach for spatial 3D calibration of lidar systems.

## 1. Introduction

The Fengyun-4 microwave detection satellite is a vital part of China’s second-generation geostationary meteorological satellite system and is the world’s first microwave remote sensing satellite in geostationary orbit. The satellite features a 5.0 m-aperture deployable primary antenna that must be deployed in orbit due to the launch vehicle’s payload constraints. However, assembly errors during deployment, combined with thermal deformation and vibration in the complex space environment, degrade the accuracy of the antenna’s surface profile, directly affecting the performance of the spaceborne microwave imager. Therefore, real-time, high-precision surface profile measurement and adaptive control are crucial.

Traditional measurement methods struggle to meet the demands of dynamic monitoring and on-orbit operations. For example, interferometry [1,2] encounters difficulties in manufacturing compensation mirrors for full-surface profile measurements. Projection measurement techniques struggle to achieve precise fringe or pattern projection due to the large distances and areas involved. Consequently, the laboratory developed a laser measurement approach and created a frequency-modulated continuous wave (FMCW) laser measurement system [3,4] for antenna surface profiling and characterization. This system integrates multiple technological advantages, including lidar and FMCW ranging [5,6]. It creatively employs equal-optical-frequency-interval resampling techniques to reduce nonlinear errors during laser frequency sweeping. It uses dual-channel synchronous counter-chirp laser ranging to correct Doppler frequency-shift errors caused by target vibrations during measurement [7,8]. The system achieves a measurement range exceeding 10 m with micrometer-level accuracy, making it suitable for microwave antenna surface-profile measurements. However, due to the complex structure of the FMCW measurement system, numerous factors affect the final measurement accuracy, including, but not limited to, system assembly errors, external environmental interference, and limitations of the measurement methodology. Therefore, improving the accuracy and reliability of the self-developed FMCW laser measurement system is a critical challenge that must be addressed urgently.

Researchers worldwide have conducted extensive work on error analysis, model development, and compensation for spatial three-dimensional calibration of laser trackers, similar to FMCW measurement systems. Since the publication of the spherical-coordinate-based laser tracker measurement system by Lau [9] et al. at the National Institute of Standards and Technology (NIST), numerous scholars have undertaken significant research on establishing system-error models and performing system calibration. Loser [10] and Muralikrishnan [11] conducted a detailed analysis of error sources in two types of laser trackers (with and without mirrors), establishing a geometric error model. They calibrated the system’s indirect error terms using length and mirror-based measurements. Hughes [12] et al. designed an optimized spatial network point layout and employed a parameter-fitting-based optimization method to enhance the robustness of geometric parameter identification, thereby improving accuracy. Conte [13] et al. developed a kinematic error model for mirrorless laser trackers using the D-H modeling method, with parameter estimation achieved through iterative optimization. Icasio-Hernández [14] et al. validated the analysis of measurement uncertainty and the optimization of geometric error parameters using network methods, providing an evaluation approach for assessing calibration accuracy in complex measurement networks. Muralikrishnan [15] et al. comprehensively compared the efficiency of network methods, length-consistency methods, and dual-face methods across various self-calibration scenarios, clarifying the advantages and disadvantages of each approach for correlation and uncertainty propagation. Similarly, domestic research has made significant strides in improving measurement accuracy. Zhang Zili [16] et al. developed a vector-based tilt-error model for mirrors, leveraging their structural characteristics to separate and compensate for errors. Song Huixu [17] proposed a two-dimensional universal joint rotary-axis system model and investigated the impact of geometric and runout errors on measurement accuracy. Cui Chengjun [18,19] et al. established error models for zero-offset, optical-axis, and vertical-axis coaxiality in femtosecond laser trackers. Xu Liang [20] et al. conducted research on geometric structure modeling and systematic error analysis of a three-dimensional laser ball-and-rod system using multibody system theory. Miao Yinxiao [21] et al. developed a geometric accuracy model with 26 error terms for FMCW lidar systems to address multiple manufacturing and assembly uncertainties. Cheng Zhi [22] et al. designed an active alignment target for laser trackers, establishing mathematical models and nonlinear correction methods to enhance measurement precision. Wang Shan [23] et al. analyzed the system’s geometric errors and calibrated and compensated them using devices such as image projectors and PSDs. While the above work offers advantages such as model completeness and universality, challenges remain in computational complexity, strong dependence on calibration fields, high coupling among multi-station parameters, and the persistence of system-level errors.

This paper presents a self-developed FMCW laser measurement system and addresses geometric, structural and optical-axis deviation errors by establishing a composite error-separation model. A calibration and verification scheme is designed to validate the effectiveness and feasibility of the proposed method, and its calibration performance is verified using experimental data. Section 2 first analyzes the system error sources and establishes a mathematical model, providing a theoretical foundation for the calibration scheme in Section 3.

## 2. Materials and Methods

### 2.1. Error Analysis and Modeling

#### 2.1.1. FMCW System Principle

The FMCW laser measurement system integrates frequency-modulated continuous-wave interferometric laser ranging with a high-precision scanning mirror to achieve three-dimensional measurement of spatial target points.

**Figure 1:**
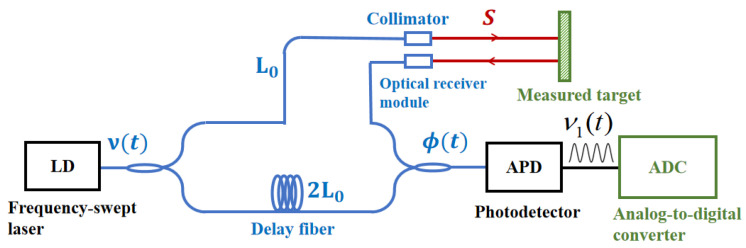
Schematic diagram of a typical swept-frequency interferometric laser ranging system.

Figure 1 shows a typical frequency-swept interferometric laser measurement system. The laser beam from the frequency-swept laser is split into two beams by a coupler. One beam serves as the local oscillator (LO) light and is transmitted through a delay fiber to the detector. The other beam serves as the measurement light, passing through an optical fiber and a collimator before illuminating the target surface. After reflection, it enters the light collection module and ultimately reaches the detector, where it interferes with the LO light. This interference is converted into an electrical signal, and the distance is determined from the frequency difference between the transmitted and reflected light.

**Figure 2:**
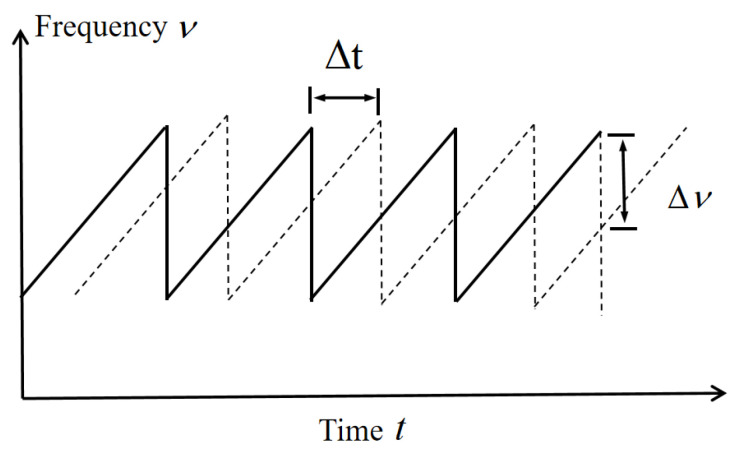
Frequency modulation waveform diagram.

The measurement light emitted by the swept-frequency laser is a frequency-modulated continuous-wave laser generated by modulating an input electrical signal. As shown in Figure 2, the system generates a sawtooth-shaped frequency signal by linearly modulating the laser’s output frequency. A frequency difference Δν arises between the measurement light and the local oscillator light due to the time delay Δt caused by the optical path difference.

Let ν(t) denote the center frequency of the laser emitted by the swept-frequency light source, w the spectral line width, c the speed of light in vacuum, and L(t) the optical path difference between the measured light and the local oscillator light. The two laser beams interfere at the detector, producing interference fringes (optical beats) with phase Φ(t). After photoelectric conversion by the detector, the instantaneous frequency ν_1_(t) of the beat-frequency electrical signal can be calculated as:

(1) $$\varphi (t)=\frac{2\pi }{c}\cdot L(t)\cdot \nu (t)$$

(2) $$\nu_{1}(t)=\frac{1}{2\pi }\cdot \frac{d\varphi (t)}{dt}=\frac{1}{c}\cdot \left[L(t)\cdot \nu^{\prime }(t)+L^{\prime }(t)\cdot \nu (t)\right]$$

Under ideal conditions, the frequency-swept laser output from the light source varies linearly. Let ν_0_ be the initial frequency of the ideal linear frequency-swept light source, and assume it begins sweeping at a constant rate ξ_0_ starting at time t = 0. Then, the center frequency of the output laser is:

(3) $$\nu (t)=\nu_{0}+\xi_{0}\cdot t$$

The measured distance is S = S_0_. The actual optical path difference between the two laser beams is twice the measured distance, i.e., L(t) = 2S_0_. After sampling the beat-frequency signal at equal time intervals and performing an FFT, the signal spectrum can be obtained. According to the Nyquist sampling theorem, to avoid spectral aliasing, the sampling frequency ν_s_ must satisfy ν_s_ ≥ 2ν_max_, where ν_max_ is the highest frequency of the beat signal. The equal optical-to-acoustic sampling frequency of 250 kHz used in this system satisfies the Nyquist condition. Ideally, the frequency corresponding to the spectrum position is the actual frequency of the beat signal, allowing the measured distance to be calculated:

(4) $$S_{0}=\frac{c}{2\cdot \xi_{0}}\cdot \nu$$

**Figure 3:**
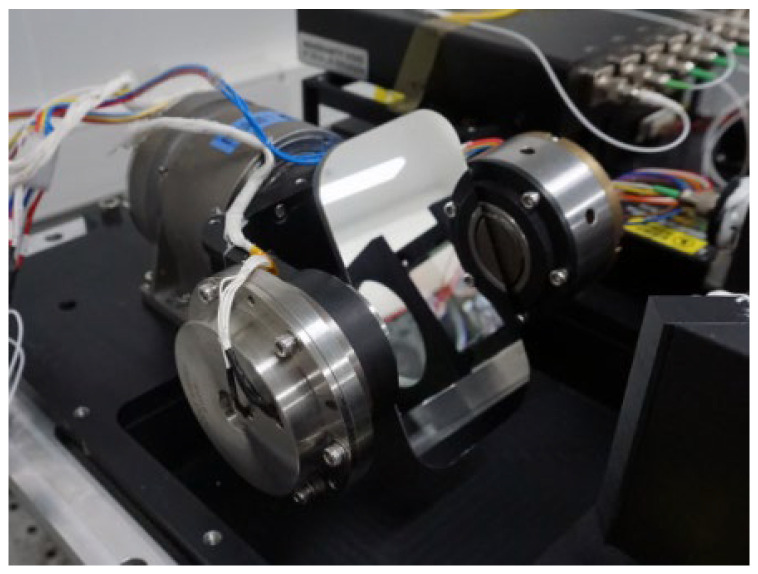
Physical photograph of the scanning mirror.

**Figure 4:**
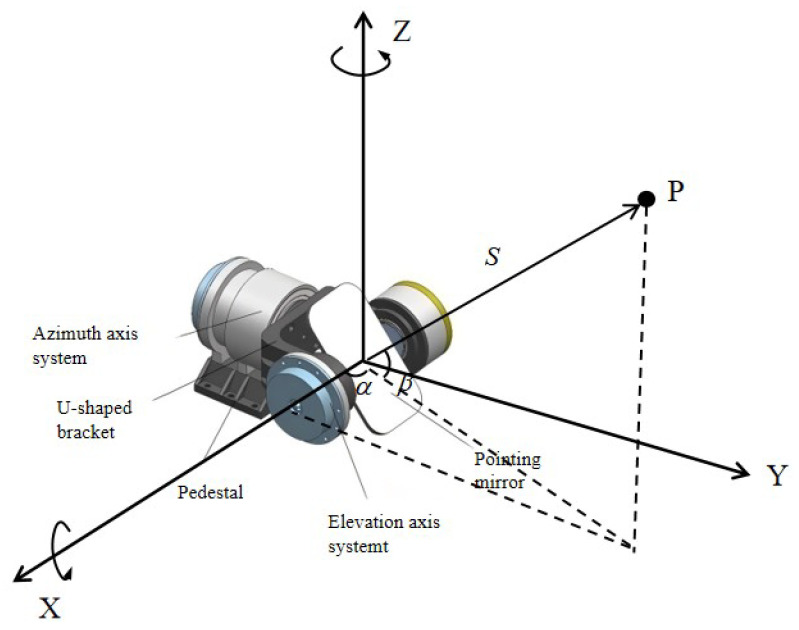
Schematic diagram of three-dimensional measurement of the scanning mirror.

The structure of the high-precision two-dimensional scanning mirror in the system (as shown in Figure 3) primarily consists of a base, U-shaped frame, azimuth axis system, elevation axis system, and pointing mirror. The axis systems employ a direct-drive solution with torque motors coupled to rotary transformers. The mirror features a center-supported structure, with the rotary transformer serving as the angular measurement module. Azimuth and elevation are statically balanced and include mechanical limiters, but they do not have locking mechanisms. During measurement, precise control of the elevation and azimuth motors enables the scanning mirror to reflect the measurement laser onto specific target points in space (as shown in Figure 4).

**Figure 5:**
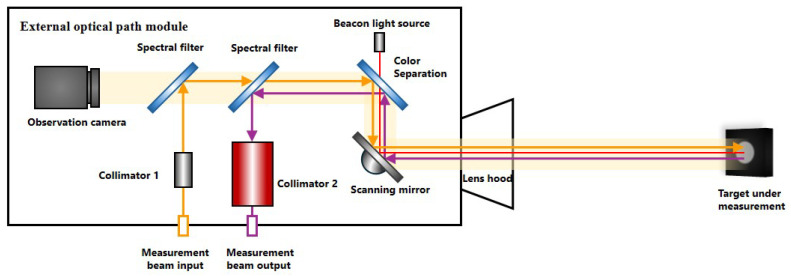
Schematic diagram of the external optical path measurement system.

During the measurement of the antenna surface profile, the approximate positions of each target point on the antenna are known. Therefore, distance measurements are obtained by precisely adjusting the scanning mirror to focus the laser on the center of each target. Combined with the three-dimensional pointing angle data, this enables reconstruction of both the antenna surface profile and the three-dimensional attitude data. Throughout the measurement process, the target points remain largely within the central field of view. The system employs a visible-light laser as the beacon light, with the beacon beam coupled to the ranging beam via a dichroic mirror. By adjusting the beacon and laser optical paths to align them coaxially and using a visible-light footprint camera to observe the beacon light’s positional deviation on the target, the closed-loop control system continuously steers the scanning mirror to precisely focus the light spot at the center of the target point. At this point, detecting the beacon light determines the actual coordinates of the target point (as shown in Figure 5).

**Figure 6:**
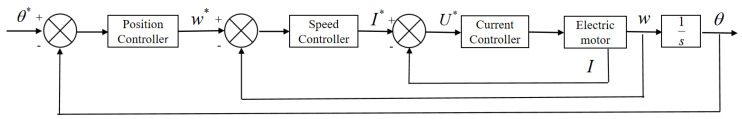
Three-loop structure of the control system.

The closed-loop control employs a three-loop PID control algorithm. Based on the imaging control mode analysis of the two-dimensional scanning mechanism, it uses position information as input to minimize positional error. The control system implements multi-loop control through a nested configuration of position, velocity, and current loops, thereby ensuring control precision (as shown in Figure 6).

#### 2.1.2. System Error Analysis

After clarifying the principle of the FMCW laser measurement system, it is necessary to analyze the primary sources of error that affect its measurement accuracy. As described above, the system employs frequency-modulated continuous-wave (FMCW) interferometric laser ranging for distance measurement and uses a high-precision scanning mirror and an angular measurement module to measure angles. To correct nonlinear errors inherent in the frequency-sweeping process of the frequency-sweeping light source, an equal optical-frequency-interval resampling technique was employed. Additionally, a dual-channel synchronous counter-sweeping laser mode was designed to correct Doppler frequency-shift errors during measurements. Parameter calibration methods were used to correct fiber dispersion errors that may arise from environmental changes or laser frequency drift. Additionally, analysis of the optical path structure outside the system space shows that errors such as rotational-axis deviation, scanning-mirror deviation, angular-measurement-module deviation [24,25], and incident-laser deviation [26] also affect the system’s measurement accuracy.

Under ideal conditions, the target point P measured by the FMCW laser measurement system can be expressed in the spatial coordinate system as:

(5) $$P=\{\begin{array}{c} x=S\cos\beta \cos\alpha \\ y=S\cos\beta \sin\alpha \\ z=S\sin\beta \end{array}$$

Here, α denotes the azimuth angle, β the elevation angle, and S the distance from the target point to the system’s zero point. The scanning mirror’s azimuth and elevation axes are orthogonal and intersect at the system’s measurement origin. Simultaneously, the measured light, after reflection by the dichroic mirror, strikes this point and is then reflected by the scanning mirror onto the target. To quantify the influence of various error parameters within the system, this section establishes the corresponding mathematical model.

##### Rotation Axis Deviation

**Figure 7:**
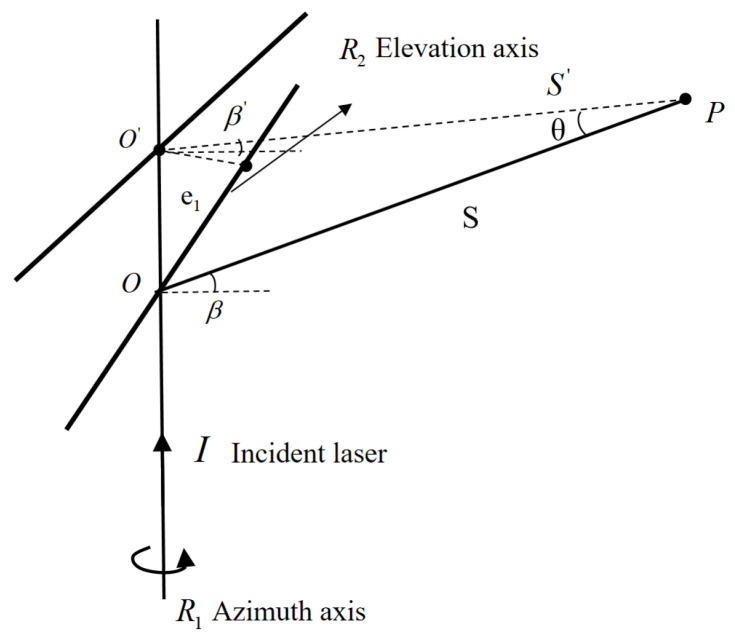
Non-coplanarity error e_1_.

**Figure 8:**
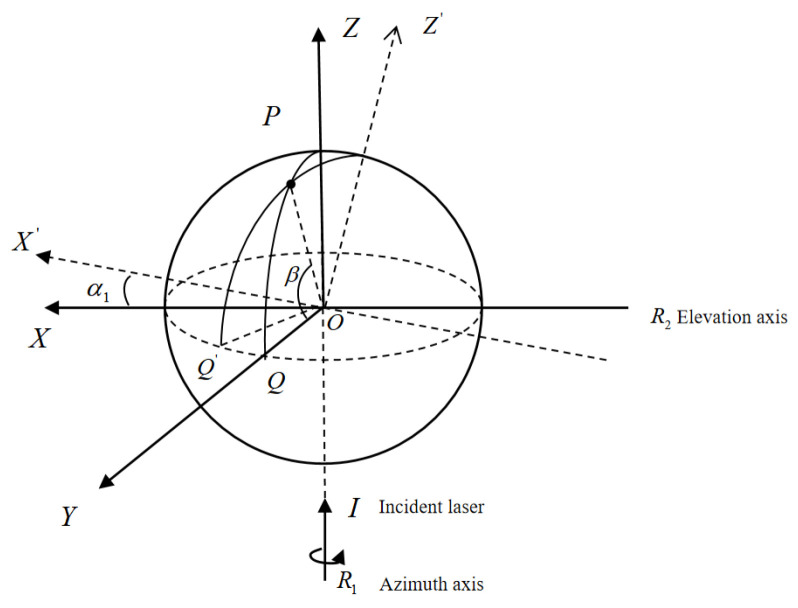
Tilt error α_1_.

Rotation axis deviation refers to the misalignment between the system’s azimuth and pitch axes. As shown in Figure 7, a non-coplanar error e_1_ exists between the azimuth axis R_1_ and the pitch axis R_2_, indicating a spatial misalignment between these axes in three-dimensional space. Furthermore, although the pitch and azimuth axes are ideally perpendicular, the actual pitch axis is inclined by an angle α_1_ from the ideal alignment (as illustrated in Figure 8), resulting in non-orthogonality between the two rotation axes.

In Figure 8, IO’P represents the ideal optical path to measurement point P, while IOP denotes the optical path when a misalignment error e_1_ exists between the two rotation axes. β and S denote the measured pitch angle and distance, respectively. As shown in Figure 7, the misalignment error e_1_ induces measurement errors in both the pitch angle β and the distance S. Using the sine rule and the small-angle approximation, we obtain:

(6) $$\sin(\Delta \beta )=\frac{e_{1}\cdot \cos\beta }{S\cdot \tan\frac{90-\beta }{2}+e_{1}\cdot \sin\beta }$$

(7) $$\Delta S=\frac{e_{1}\cdot (1-\sin\beta )}{\tan\frac{90-\beta }{2}}=e_{1}\cdot \cos\beta$$

When the pitch and azimuth axes are not orthogonal, Figure 8 shows that the true coordinate system X’YZ’ is obtained by rotating the coordinate system XOZ about the *Y*-axis by angle α_1_. The arc length QQ’ represents the effect of the inclination angle α_1_ on the azimuth angle. The azimuth error can be expressed as:

(8) $$\Delta \alpha =\alpha_{1}\cdot \tan\beta$$

Among these, ∆α, ∆β, and ∆S represent the deviations in elevation angle, azimuth angle, and distance, respectively. α, β, and S denote the azimuth angle, elevation angle, and distance measured by the system.

##### Scanning Mirror Deviation

**Figure 9:**
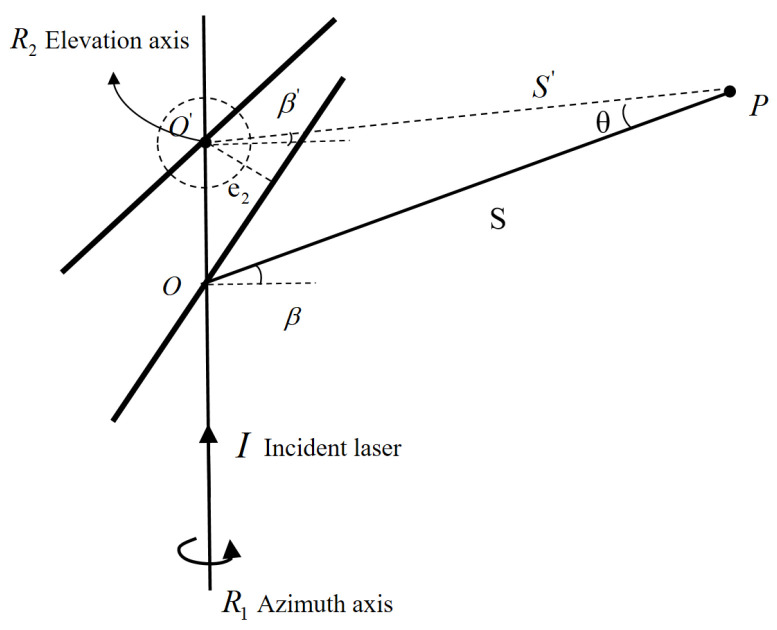
Non-coplanarity error e_2_.

**Figure 10:**
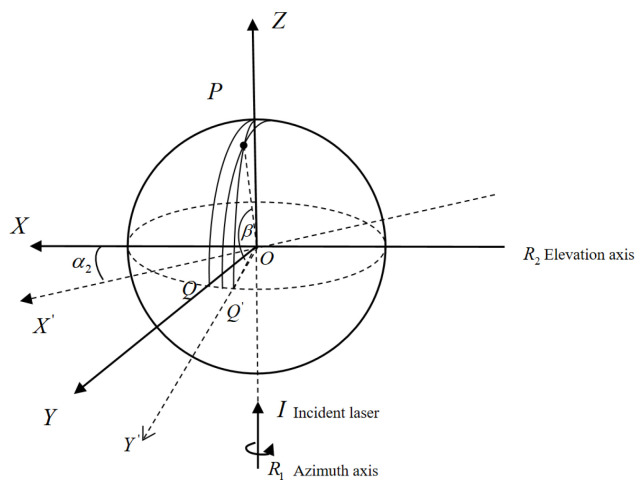
Tilt error α_2_.

Scanner misalignment comprises both the plane deviation error and the angular tilt error relative to the pitch axis. The plane deviation error, e_2_, quantifies how far the scanner deviates from the pitch axis, as shown in Figure 9. The angular tilt error, indicated by the tilt angle α_2_, describes the scanner’s tilt relative to the pitch axis, as depicted in Figure 10. Together, these errors highlight the scanner’s deviation from the pitch axis.

In Figure 10, for target point P, the ideal optical path is IO’P, where S’ and β’ denote the ideal distance and pitch angle of the measured point. When a plane error e_2_ is present, the IOP becomes the actual optical path, yielding the measured distance S and pitch angle β. Through calculation, we obtain:

(9) $$\sin(\Delta \beta )=\frac{e_{2}\cdot \cos\beta }{S\cdot \sin\frac{90-\beta }{2}+e_{2}\cdot \sin\beta }$$

(10) $$\Delta S=\frac{e_{2}\cdot \left(1-\sin\beta \right)}{\sin\frac{90-\beta }{2}}={2e}_{2}\cdot \sin\frac{90-\beta }{2}$$

Under ideal conditions, the scanning mirror is parallel and coincident with the pitch axis, and its normal points toward OY. When an inclination angle α_2_ exists, the mirror’s normal points toward OY’, equivalent to rotating the XYZ coordinate system by angle α_2_ about the *Z*-axis. Analysis shows that the tilt angle α_2_ introduces errors in azimuth measurements. Calculations indicate:

(11) $$\Delta \alpha =\frac{\alpha_{2}}{\cos\beta }$$

##### Incident Laser Deviation

In addition to deviations in the rotation axis and scanning mirror, the laser’s offset is another major source of measurement errors. If the incident laser is not aligned coaxially with the vertical optical axis, the point of impact will shift away from the scanning mirror’s rotational center, affecting measurement accuracy. When calculating the incident laser offset error, two displacements are important: the tilt angle θ between the laser and the vertical axis, and the translational offset T between the laser and the vertical axis.

**Figure 11:**
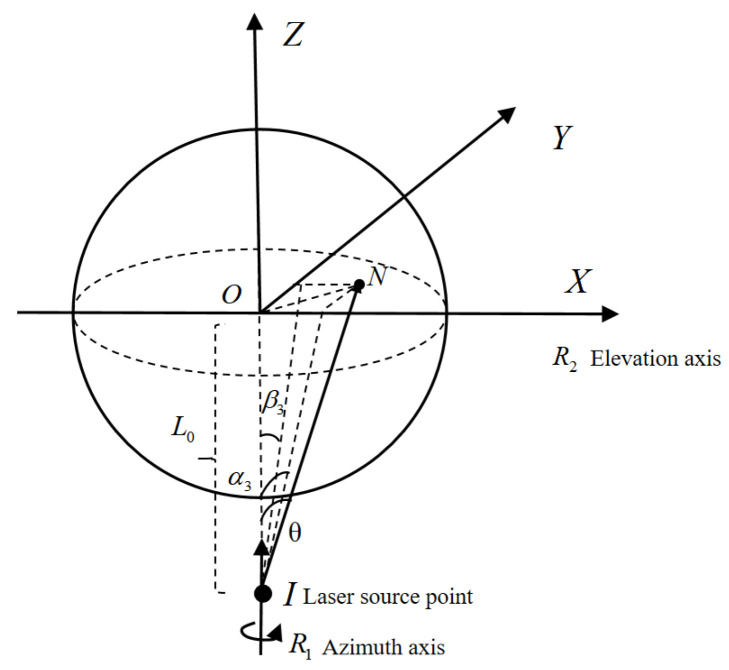
Tilt offset θ.

As shown in Figure 11, the laser’s tilt angle θ can be decomposed into the tilt angle α_3_ in the XOZ plane and the tilt angle β_3_ in the YOZ plane. Here, L_0_ denotes the ideal distance from the reflection point on the dichroic mirror to the rotational center of the scanning mirror. Tilt angles affect the system’s angular measurements. The calculations yield:

(12) $$\Delta \alpha =\frac{L_{0}\cdot \alpha_{3}}{S\cdot \cos\beta }$$

(13) $$\Delta \beta =\frac{L_{0}\cdot \beta_{3}}{S-L_{0}\cdot \beta_{3}\cdot \cot\left(\frac{90-\beta_{3}-\beta }{2}\right)}$$

**Figure 12:**
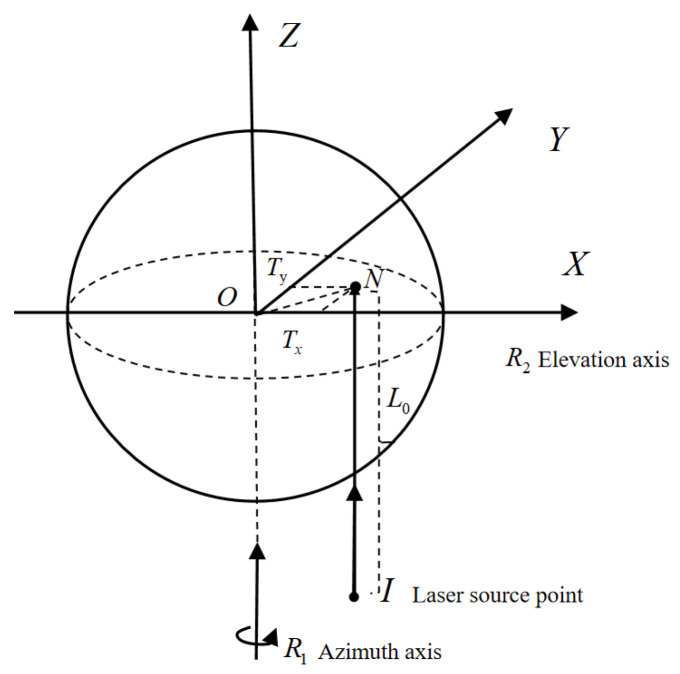
Translation offset T.

As shown in Figure 12, the translation offset of the incident laser refers to its parallel but non-coincident alignment with the vertical axis. The translation offset T can be decomposed into the offset T_x_ in the XOZ plane and the offset T_y_ in the YOZ plane. Calculations indicate that the effect of the translation offset on angular measurement accuracy is as follows:

(14) $$\sin(\Delta \alpha )=\frac{T_{x}}{S\cdot \cos\beta }$$

(15) $$\tan(\Delta \beta )=\frac{T_{y}}{S+T_{y}\cdot \cot(\frac{90-\beta }{2})}$$

##### Angle Measurement Module Deviation

Because the system uses a rotary transformer as the angular measurement module to measure the target’s pitch and azimuth angles, it is necessary to account for the effect of the rotary transformer’s inherent eccentricity error on the accuracy of angle measurements. Let O’denote the geometric center of the rotor of the synchro, and O denote the ideal rotational axis. When these two points are not coincident, eccentricity error occurs. Establish a Cartesian coordinate system with origin O. Assume the eccentricity components e_x_ and e_y_ of the rotor’s geometric center O’relative to the rotational axis O in the x and y directions, respectively, and the effective working radius R of the rotary encoder. When the rotor rotates through the actual angle α, due to eccentricity, the actual position of the measurement signal detection point is:

(16) $$P=(R\cdot \cos\alpha +e_{x},\;R\cdot \sin\alpha +e_{y})$$

Since the eccentricity is generally much smaller than the working radius, applying a Taylor series expansion to the formula and setting Ax = e_x_/R and Ay = e_y_/R yields the azimuth angular measurement error caused by eccentricity as:

(17) $$\Delta \alpha =A_{y}\cdot \sin\alpha -A_{x}\cdot \cos\alpha$$

Similarly, the angular eccentricity error in the pitch angle can be expressed as:

(18) $$\Delta \beta =E_{y}\cdot \sin\beta -E_{x}\cdot \cos\beta$$

In summary, a systematic analysis was conducted of the rotational axis deviation, scanning mirror misalignment, incident laser deviation, and angular measurement module deviation of the FMCW laser measurement system, covering the primary geometric errors in the external optical path structure. Under the small-error assumption (i.e., each error parameter satisfies e_i_/S ≪ 1 relative to the measurement distance S, and the angular error satisfies the small-angle approximation), this paper employs a first-order linear superposition model for error modeling. To assess the reasonableness of neglecting higher-order terms, an order-of-magnitude analysis was conducted on the second-order coupling terms of the error parameters. The analysis showed that each second-order coupling term is approximately 0.1% to 2% of the first-order terms, making them negligible. For scenarios where the measurement angle exceeds 30° or where higher precision is required, the primary second-order coupling terms (e_1_ with α_1_ and e_2_ with α_2_) were incorporated into the model. The overall error model for the final system can be expressed as:

(19) $$\alpha_{4}=\alpha +\alpha_{1}\cdot \tan\beta +\frac{\alpha_{2}}{\cos\beta }+\frac{L_{0}\cdot \alpha_{3}}{S\cdot \cos\beta }+\arcsin(\frac{T_{x}}{S\cdot \cos\beta })+A_{y}\cdot \sin\alpha -A_{x}\cdot \cos\alpha$$

(20) $$\begin{array}{l} \beta_{4}=\beta +E_{y}\cdot \sin\beta -E_{x}\cdot \cos\beta +\arcsin(\frac{e_{1}\cdot \cos\beta }{S\cdot \tan(\frac{90-\beta }{2})+e_{1}\cdot \sin\beta })+\\ \arcsin(\frac{e_{2}\cdot \cos\beta }{S\cdot \sin(\frac{90-\beta }{2})+e_{2}\cdot \sin\beta })+\frac{L_{0}\cdot \beta_{3}}{S-L_{0}\cdot \beta_{3}\cdot \cot(\frac{90-\beta_{3}-\beta }{2})}+\arctan(\frac{T_{y}}{S+T_{y}\cdot \cot(\frac{90-\beta }{2})}) \end{array}$$

(21) $$S_{4}=S+e_{1}\cdot \cos\beta +2e_{2}\cdot \sin(\frac{90-\beta }{2})+\frac{e_{1}\cdot \alpha_{1}\cdot \cos\beta }{\tan(\frac{90-\beta }{2})}+\frac{e_{2}\cdot \alpha_{2}\cdot \cos\beta }{\sin(\frac{90-\beta }{2})}$$

**Figure 13:**
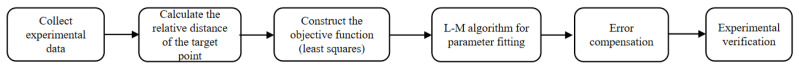
Calibration flowchart.

This error model contains 13 parameters to be calibrated: [L_0_, e_1_, α_1_, e_2_, α_2_, α_3_, β_3_, T_x_, T_y_, A_x_, A_y_, E_x_, E_y_]. Here, S, α, and β denote the measured distance, azimuth angle, and elevation angle, respectively, while the ideal distance, azimuth angle, and elevation angle are denoted as S_4_, α_4_, and β_4_. As shown in the calibration process in Figure 13, after completing parameter fitting, the corrected ideal values can be obtained by inputting the measured values, substituting the derived 13 error parameters, and computing the correction factors.

Based on the established error parameter model described above, error propagation theory is used to quantitatively analyze the system’s measurement errors. For a measurement function $f(p_{1},p_{2},\ldots p_{n})$ influenced by multiple independent error sources, its combined standard uncertainty is calculated using the following formula:

(22) $$\delta_{f}=\sqrt{\sum_{i=1}^{n}{\left(\frac{\partial f}{\partial p_{i}}\right)}^{2}\times {\delta_{p_{i}}}^{2}}$$

where $\partial f/\partial p_{i}$ is the sensitivity coefficient, representing the influence of the i-th error source on the measurement result, and $\delta_{p_{i}}$ is the standard uncertainty of the i-th error parameter. According to the distance correction formula in the overall error model, perform a total differentiation of S_4_:

(23) $$\sigma_{S_{4}}^{2}=\sigma_{S}^{2}+{\left(\frac{\partial S_{4}}{\partial e_{1}}\right)}^{2}\cdot \sigma_{e_{1}}^{2}+{\left(\frac{\partial S_{4}}{\partial e_{2}}\right)}^{2}\cdot \sigma_{e_{2}}^{2}+{\left(\frac{\partial S_{4}}{\partial \alpha_{1}}\right)}^{2}\cdot \sigma_{\alpha_{1}}^{2}+{\left(\frac{\partial S_{4}}{\partial \alpha_{2}}\right)}^{2}\cdot \sigma_{\alpha_{2}}^{2}$$

By applying the total differential to the azimuth angle α_4_ correction formula, we obtain:

(24) $$\sigma_{\alpha_{4}}^{2}=\tan^{2}\beta \cdot \sigma_{\alpha_{1}}^{2}+\frac{1}{\cos^{2}\beta }\cdot \sigma_{\alpha_{2}}^{2}+\frac{L_{0}^{2}}{S^{2}\cdot \cos^{2}\beta }\cdot \sigma_{\alpha_{3}}^{2}+\frac{1}{S^{2}\cdot \cos^{2}\beta }\cdot \sigma_{T_{x}}^{2}+\cos^{2}\alpha \cdot \sigma_{Ax}^{2}+\sin^{2}\alpha \cdot \sigma_{Ay}^{2}$$

Taking the total differential of the pitch angle β_4_ correction formula yields: (where C_e1_ and C_e2_ are the sensitivity coefficients of the pitch angle with respect to the dihedral error)

(25) $$\sigma_{\beta_{4}}^{2}=\cos^{2}\beta \cdot \sigma_{E_{x}}^{2}+\sin^{2}\beta \cdot \sigma_{E_{y}}^{2}+\frac{L_{0}^{2}}{S^{2}}\cdot \sigma_{\beta_{3}}^{2}+\frac{1}{S^{2}}\cdot \sigma_{T_{y}}^{2}+C_{e_{1}}^{2}\cdot \sigma_{e_{1}}^{2}+C_{e_{2}}^{2}\cdot \sigma_{e_{2}}^{2}$$

Since spherical coordinates were ultimately converted to the Cartesian coordinate system (x, y, z) during the calculation of spatial coordinates and the point cloud measurement of the antenna surface, the total differential was computed for the x, y, and z coordinates, respectively. The total error for the three-dimensional point positions is thus obtained as:

(26) $$\sigma_{3D}=\sqrt{\sigma_{x}^{2}+\sigma_{y}^{2}+\sigma_{z}^{2}}=\sqrt{\sigma_{S_{4}}^{2}+S_{4}^{2}\cdot \cos^{2}\beta_{4}\cdot \sigma_{\alpha_{4}}^{2}+S_{4}^{2}\cdot \sigma_{\beta_{4}}^{2}}$$

Through the error model analysis, the expressions of sensitivity coefficients are shown in Table 1.

The specific values of the sensitivity coefficients and the calculation of the system’s combined measurement uncertainty will be presented in Section 4.

#### 2.1.3. Simulation Model

**Figure 14:**
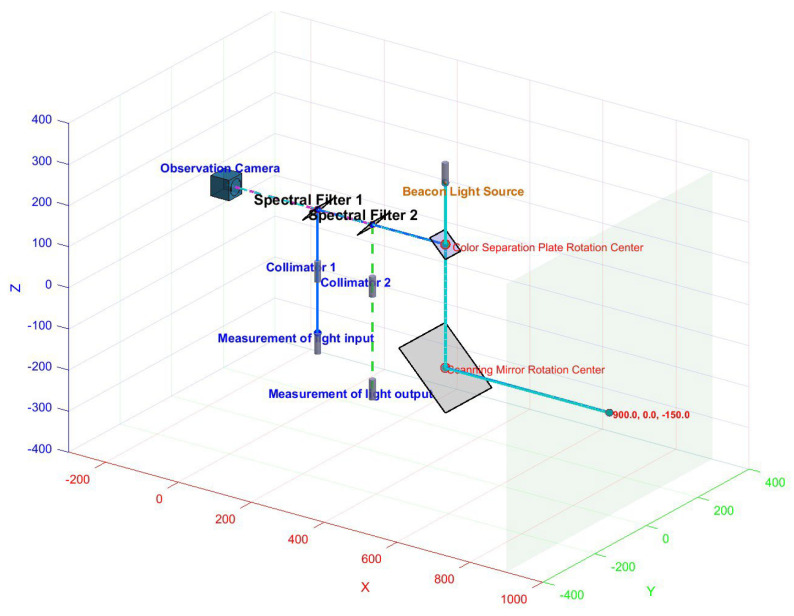
System simulation diagram.

To validate the correctness of the aforementioned theoretical derivation and to visually demonstrate the impact of each error parameter on the system’s measurement accuracy, a simulation model was developed for the system, as shown in the figure:

Figure 14 illustrates the geometric configuration of the simulation system. The simulation model comprises: a laser light source with a color-splitting lens assembly, where translation offsets (T_x_, T_y_) and laser tilt errors (α_3_, β_3_) can be configured; a dual-axis scanning mirror: independent settings for the azimuth and elevation axes’ misalignment errors (e_1_, e_2_) and tilt errors (α_1_, α_2_), along with the target plane. Simulation is achieved through inverse solution: Given the spatial coordinates of the target point, the required scanning angles (α_0_, β_0_) are calculated based on ideal geometric relationships. Error parameters are then introduced to obtain the actual scanning angles (α, β) and the measured distance value S. Finally, the deviation between the measured point and the target point is calculated.

**Figure 15:**
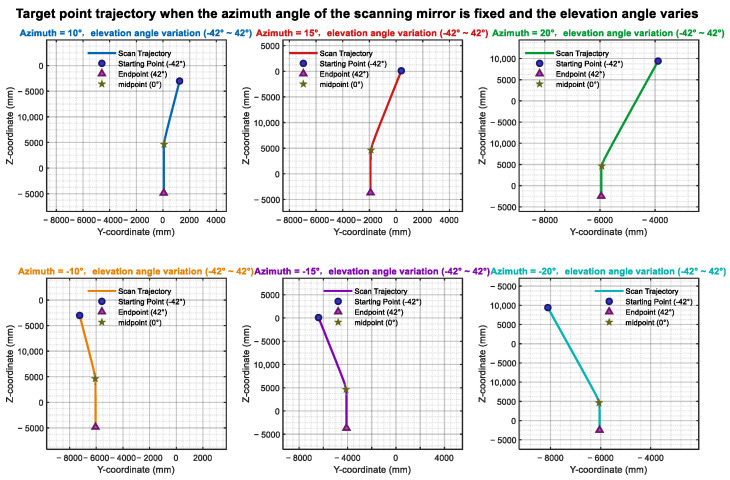
Azimuth angle tilt trajectory variation.

**Figure 16:**
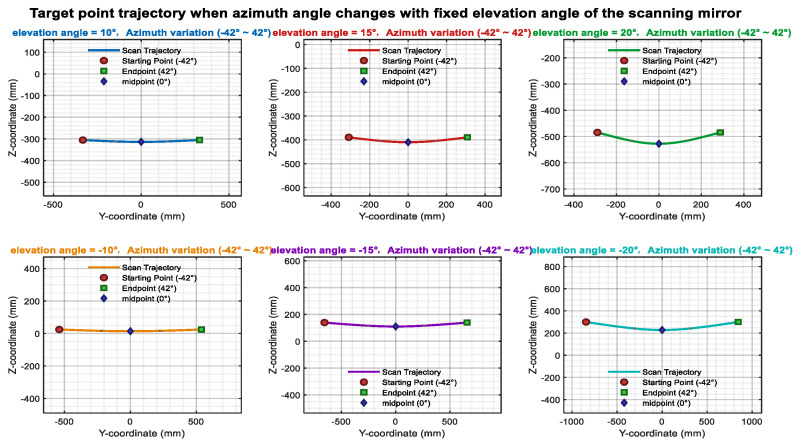
Elevation angle tilt trajectory variation.

Figure 15 illustrates the influence of the azimuth axis tilt error α_1_ on the scanning trajectory. Ideally, when β is held constant, and only α is varied, the laser trace on the target plane should form a straight line. Upon introducing the tilt error α_1_, the laser trajectory progressively deviates from linearity as α_1_ increases. Similarly, Figure 16 presents an analysis of the elevation axis tilt error α_2_ and its impact on the scanning trajectory.

**Figure 17:**
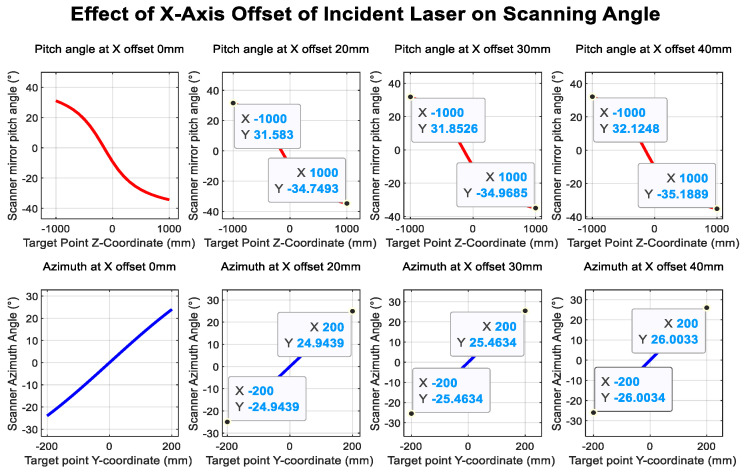
Incident laser offset in the *x*-axis direction.

**Figure 18:**
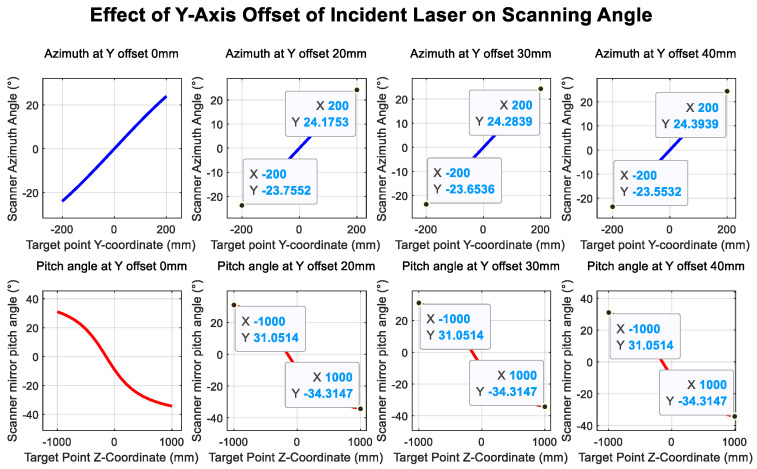
Incident laser tilt in the *y*-axis direction.

Figure 17 and Figure 18 illustrate the influence of translational offsets in the X- and Y-directions of the incident laser beam, respectively. As shown in the curves, the laser translational offset significantly affects the accuracy of both angle and distance measurements.

**Figure 19:**
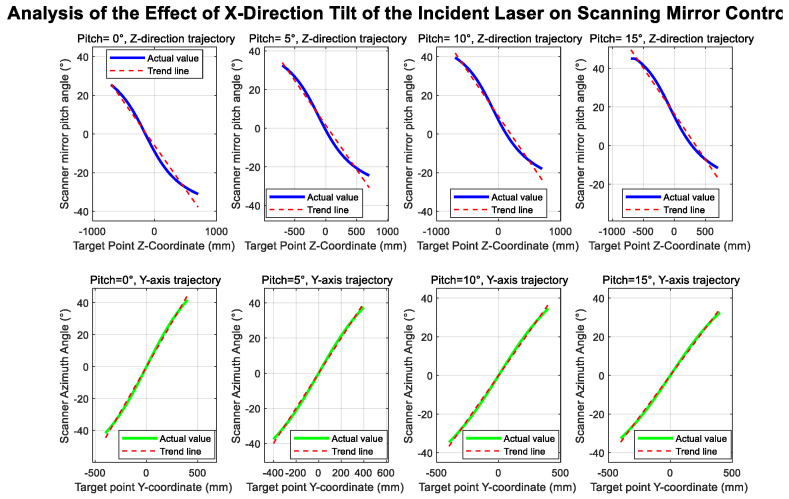
Incident laser tilt in the *x*-axis direction.

**Figure 20:**
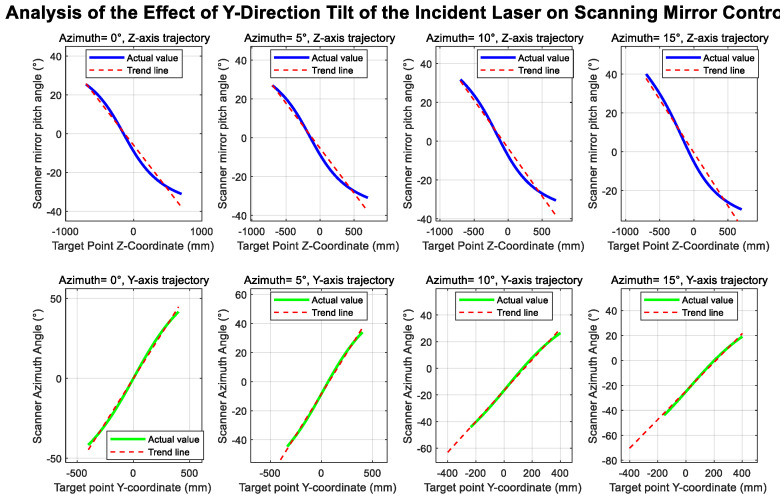
Incident laser offset in the *y*-axis direction.

Figure 19 and Figure 20 show the deviations in the scanning mirror’s elevation and azimuth angles from their ideal values as the laser moves from target point 1 to target point 2, caused by tilt error. The results indicate that rotation-axis tilt error and translational offset are the dominant factors affecting angle measurement accuracy.

Through theoretical derivation and simulation, this chapter establishes an error propagation model for the FMCW system and identifies the critical error parameters that require calibration. Because the specific values of these parameters depend on the system’s assembly state and cannot be determined analytically, experimental calibration is necessary. A dedicated calibration methodology will be developed in the following chapter.

### 2.2. Experimental Design and Implementation

#### 2.2.1. Selection of Calibration Method

Numerous 3D spatial calibration techniques are available for lidar systems, each with different site, accuracy, and complexity requirements. Since the FMCW system needs simultaneous calibration of geometric-structure and optical-axis-offset errors, choosing an effective calibration method is essential. Several standard laser-system calibration methods, such as linear-constraint calibration, spatial fixed-plane constraint [27], sphere-array calibration, and planar calibration, were reviewed. Considering the laboratory facilities and equipment, a hybrid approach combining linear-constraint and planar calibration methods was selected, leveraging high-precision reference standards as benchmarks during calibration.

Once the calibration method is chosen, select an error-correction approach. The most common correction strategies for laser system calibration are divided into two types: coordinate error correction and spatial distance correction. The spatial distance correction method is preferred here. This approach does not depend on an absolute coordinate system; instead, it uses only the known relative distances between target points within the calibration area as constraints. Given two target points i and j on the reference gauge with known relative distance L_ij_, the FMCW system measures these points, and the inter-point distance d_ij_(δ) is computed using the error model from Section 2. An objective function to minimize the distance residual is then formulated.

(27) $$\min\sum_{ij}{\left[d_{ij}(\delta )-L_{ij}\right]}^{2}$$

Among these, δ denotes the error parameter vector that must be determined. This method demonstrates excellent error isolation. In FMCW systems, measurement errors primarily arise from internal geometric inaccuracies and optical-axis offset errors. The spatial ranging correction method calibrates internal system parameters using relative distance constraints, thereby preventing external factors from interfering with calibration. Depending on application needs, the primary use of FMCW systems is to measure antenna surface profiles. The key metric for profile evaluation is the relative spatial relationships among points on the antenna surface (e.g., the RMS surface profile accuracy), not absolute coordinate values. The spatial ranging correction method precisely ensures the accuracy of relative distances between points, fully aligning with the application objective. Therefore, this paper selects the spatial ranging correction method as the error-correction strategy, solving for parameters using known relative distances as constraints.

Although single-station measurement methods can collect observational data, they suffer from insufficient observational redundancy and severe parameter coupling. To address these problems, a multi-station measurement approach is adopted, in which multiple stations measure the same set of target points. By altering the relative pose between the system and the targets (including translation and rotation), the distinguishability of parameters is enhanced. In summary, the calibration scheme adopted in this paper is defined as follows: System calibration is achieved by employing a planar calibration method based on high-precision reference scales, combined with a spatial ranging correction strategy and a multi-station measurement calibration framework.

#### 2.2.2. Experimental Design and Data Acquisition

To ensure the reliability and repeatability of the calibration experiment, a high-precision calibration platform was constructed. The experimental system comprises the following high-precision equipment: an FMCW laser radar ranging system, a high-precision reference scale (accuracy: ±1 μm/m), micro-bead targets, a PI stage, an electronic level, etc. During the experiment, an FMCW laser measurement system was used to measure the target point. The position of the beacon light was observed via the footprint camera, and the scanning mirror was controlled to center the measurement beam on the target point.

The data acquisition process is divided into two phases: First, a zero-position calibration experiment is conducted. In addition to the difference in fiber length between the local oscillator optical path and the measurement optical path, the measurement optical path also passes through spatial optical components, such as lenses and collimators, before being emitted through the scanning mirror. Therefore, besides the measurement distance 2S, there is an additional length difference between the measurement optical path and the local oscillator optical path caused by these components. Thus, it is first necessary to calibrate this difference to determine the system’s initial geometric reference. Then, a multi-station measurement experiment is conducted to obtain observation data for parameter fitting.

**Figure 21:**
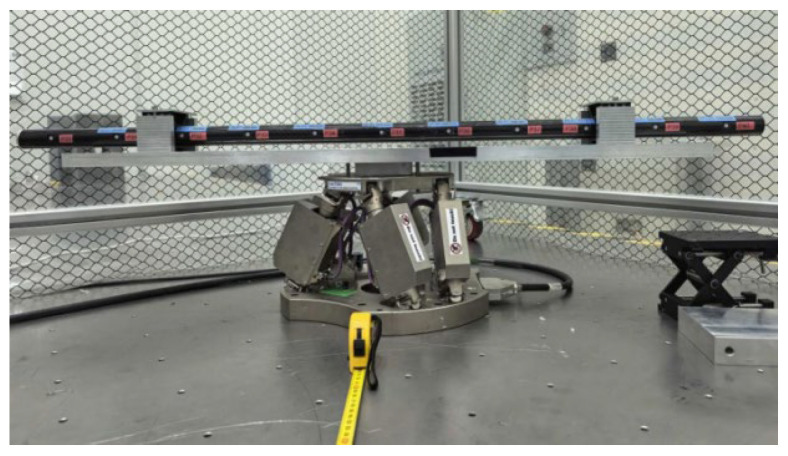
Schematic diagram of reference gauge placement.

**Figure 22:**
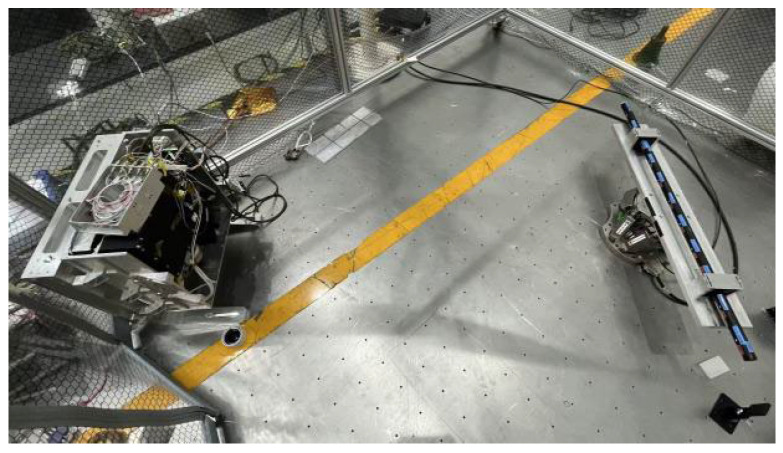
Schematic diagram of the system measurement.

**Figure 23:**
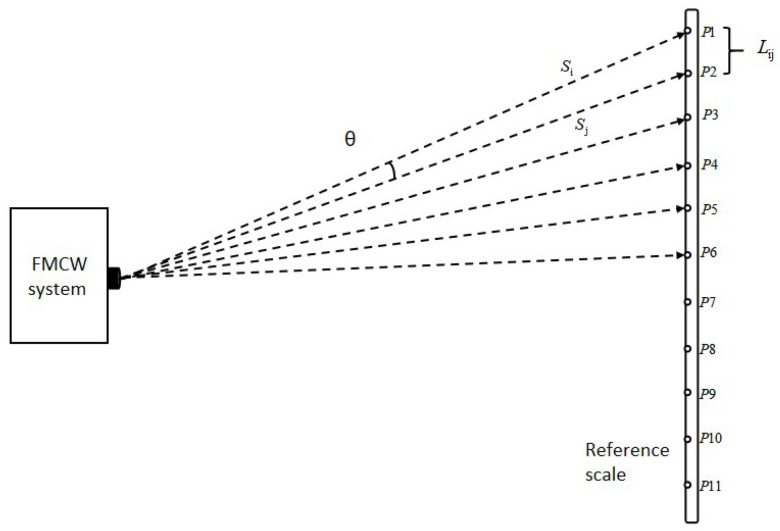
Schematic diagram of zero-position calibration.

First, the reference scale is positioned as shown in Figure 13, with 11 target points distributed linearly in one dimension. The FMCW system is installed approximately 2.3 m from the reference scale (see Figure 21, Figure 22 and Figure 23).

From the simulation analysis, it can be observed that when the scanning mirror’s azimuth and elevation angles are tilted, moving only the azimuth or elevation angle results in a curved laser trajectory. Therefore, during the system’s zero-position calibration, it is necessary to continuously adjust the azimuth and elevation angles while observing changes in the trajectory pattern. Ideally, when the zero positions of the elevation axis and azimuth axis are accurate, the scanning trajectory should be a straight line when β is fixed and only α is rotated. While ensuring that the trajectory pattern remains linear, data are collected by sequentially aiming at the 11 target points, thereby achieving zero-position calibration. Table 2 shows the position information of the 11 target points on the reference ruler. To increase data redundancy, the PI stage is used to translate the reference scale along the Y direction (perpendicular to its length), and measurements are repeated at three positions: Y = 0 mm, −10 mm, and −20 mm.

After completing the zero-position calibration of the FMCW system optical path, the multi-station measurement method is used to measure the planar target points and obtain the original observation data of the target points, thereby solving the geometric structure error and beam offset error parameters in the error model established in Section 2.

**Figure 24:**
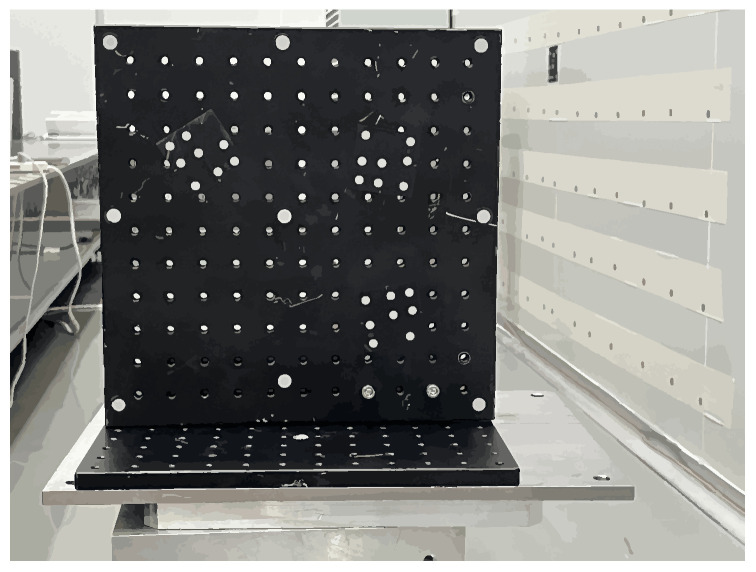
Planar target diagram.

**Figure 25:**
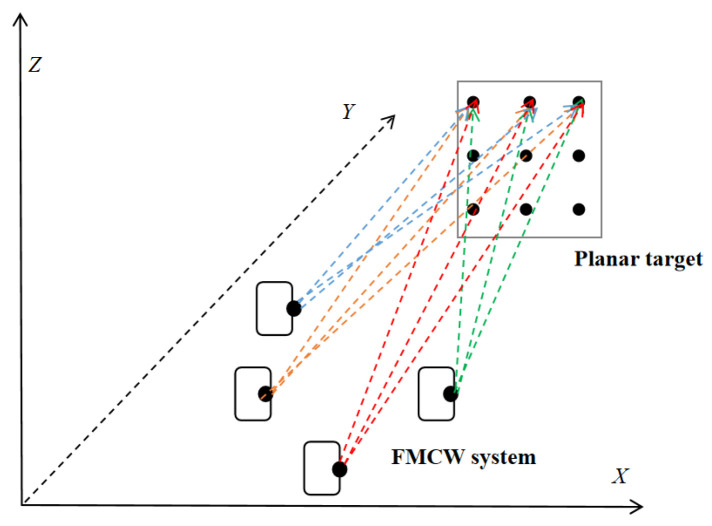
Schematic diagram of multi-station measurements.

First, secure the planar target to the platform, ensuring it is level (calibrated using an electronic level). Next, mount the FMCW measurement system on the PI stage, positioned approximately 2.5 m directly in front of the target. Control the PI stage to move the system to seven distinct measurement stations (S_1_, S_2_, S_3_…S_7_). The reference target points are arranged in a 3 × 3 grid (as shown in the Figure 24). The positions of these target points are determined by a high-precision laser tracker (accuracy < 50 μm):

Table 3 shows the location information of the nine planar target points. At each station, observe the measurement beam from the footprint camera control system sequentially illuminate the center of each target point on the plane (as shown in the Figure 25). Repeat the measurement 20 times for each target point, recording its raw distance value (S), azimuth angle (α), and elevation angle (β). Calculate the mean and standard deviation for each target point, then remove outliers. These values will be used for subsequent parameter solutions and to evaluate the repeatability of the measurement results.

## 3. Results

According to the zero-position calibration scheme designed in Section 2.2.2, measurements were conducted on 11 target points on the reference scale at three positions: Y = 0 mm, −10 mm, −20 mm. Taking the Y = 0 mm position as an example, Table 4 presents variations in the azimuth angle and ranging values with target-point positions, assuming the elevation angle is maintained at −2.0601°.

Through the above measurement data and the known relative positions between target points, the system zero-position distance L is calculated using the law of cosines for triangulation:

(28) $$L_{ij}^{2}={\left(S_{i}-L\right)}^{2}+{\left(S_{j}-L\right)}^{2}-2\cdot \cos(\alpha_{i}-\alpha_{j})\cdot (S_{i}-L)\cdot (S_{j}-L)$$

where S_i_ and S_j_ are the distances measured by the system, α_i_ and α_j_ are the corresponding azimuth angles, and L_ij_ is the reference distance between target points. By calculating and averaging multiple sets of experimental data, the actual system zero-position point L is determined to be 2.302 m. In subsequent use of the measurement system to obtain target-point ranging values, it is necessary to subtract the zero-position value from the measured distance values before performing the fitting calibration. The structural error parameters of the system are determined from the measured data, combined with the error-parameter model established in Section 2.

**Figure 26:**
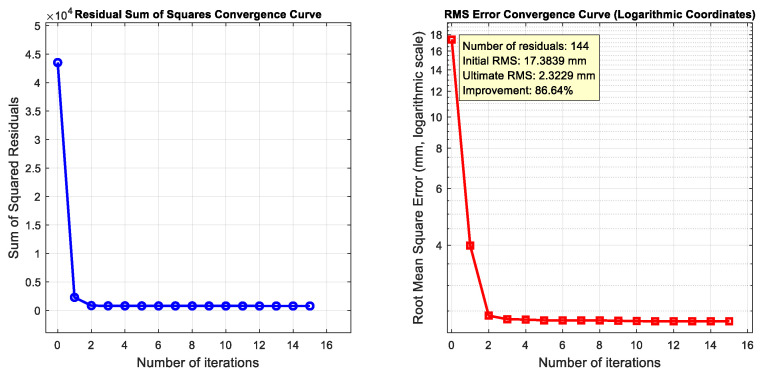
Residual convergence curve during the optimization process.

After completing the zero-position calibration, data were acquired for nine planar target points using the seven-station measurement scheme described in Section 2.2.2. The data for Station 1 are shown in the Table 5:

Based on the parameter-error-propagation equation established in Section 2, an optimization objective function for space-distance correction is constructed. During the data analysis process, the first four station data sets (S_1_~S_4_) are used for fitting, yielding a total of 144 distance constraints, and the L-M (Levenberg–Marquardt) algorithm is employed to obtain the solution. Figure 26 shows the objective function values during the iterative optimization process. As the number of iterations increases, the function values continuously decrease and eventually stabilize.

**Figure 27:**
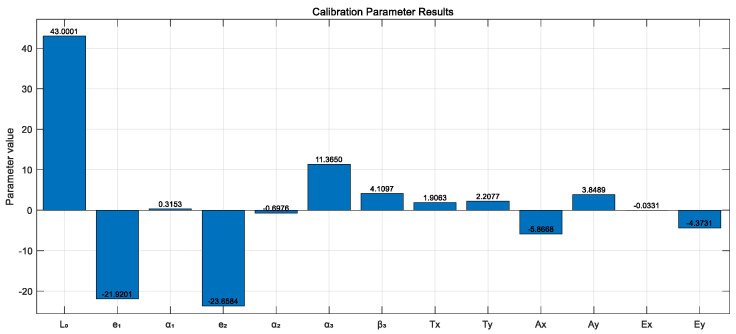
Calibration results of error parameters.

The system error parameters obtained through the final fitting solution are shown in Figure 27.

To evaluate the calibration method’s generalization capability, three independent operating conditions (S_5_–S_7_) were used for validation experiments. These three conditions were not utilized during parameter estimation. Unlike the data from the first four conditions, the data for these three conditions were acquired at different rotation angles of the system.

**Figure 28:**
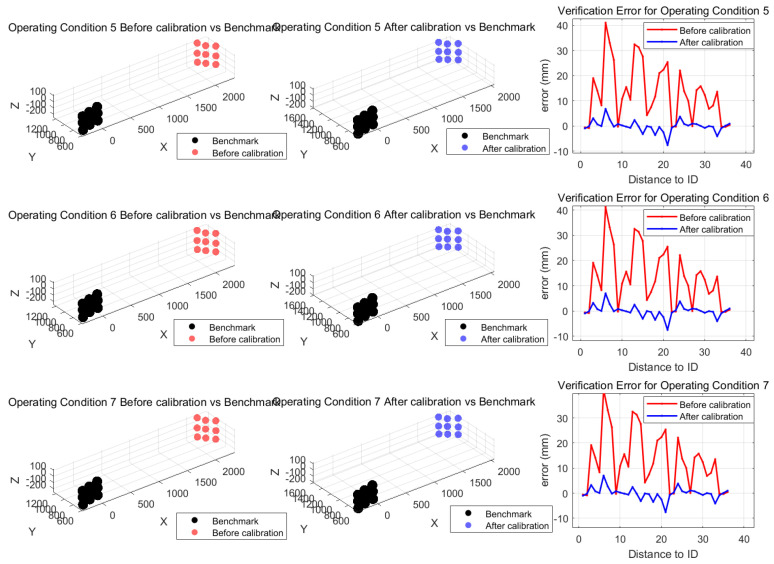
Comparison of point cloud spatial distributions under validation conditions.

**Figure 29:**
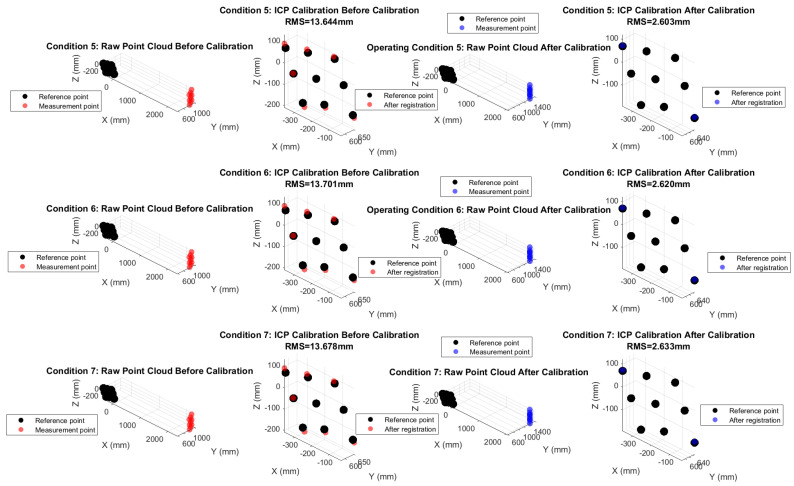
Comparison of point cloud registration results.

The comparison above shows that the spatial distribution of the point cloud under pre-calibration conditions differs significantly from that of the reference data (as shown in the Figure 28 and Figure 29). If measurements of the target points on the antenna were taken with the pre-calibration system, it would be impossible to accurately map the antenna surface profile. After parameter fitting and system calibration, the relative distance between two points under operating conditions S_5_ to S_7_ improved significantly over the baseline, with the RMS error reduced from 17.5 mm to approximately 2.3 mm. The ICP registration residuals for the spatial point cloud decreased from an average of 13.7 mm to approximately 2.5 mm, with a marked improvement in shape retention. Compared with pre-calibration, shape similarity improved by 86.6%. Using the calibrated point cloud to describe the antenna surface profile would significantly increase accuracy, demonstrating the effectiveness of this method.

In Section 2, we employed error propagation theory to conduct a quantitative analysis of systematic errors and derive expressions for each sensitivity coefficient. Using experimental test data, calibrated error parameters, calibration residual values, and the number of data points, we can calculate each error source’s contribution to the measurement uncertainty (as shown in the Table 6).

As indicated in the table above, the uncertainty in the distance measurement, σ_S4_ = 0.251 mm, primarily arises from the rotation-axis misalignment error e_1_ and the scanning mirror misalignment error e_2_. The azimuth uncertainty, σ_α4_ = 1391″ (around 0.39°), is primarily caused by the angular measurement eccentricity error Ax and the laser incidence tilt error α_3_. The azimuth angle uncertainty σ_β4_ = 307″ (around 0.085°) mainly results from the laser incidence tilt error β_3_. The overall three-dimensional position uncertainty σ_3D_ = 17.2 mm represents about 0.68% of the measured distance S. Each component’s uncertainty is much smaller than its respective measurement parameter. This demonstrates the ability to effectively describe the primary sources of error in the system, with each error parameter’s influence remaining within a controllable range.

Secondly, the reference gauge used in the experiment had an accuracy of about 1 μm. In contrast, the reference data for the planar target were more precise, with an error of less than 50 μm. As a result, the reference gauge error contributed only 0.04% to the overall system error, while the error in the target reference data accounted for approximately 2.1%. Both values were significantly smaller than the system’s residual error. This indicates that the accuracy of the reference data met the requirements of the calibration experiment and did not significantly affect the reliability of the calibration results. To ensure the system’s accuracy and applicability across different measurement distances, potential nonlinearities were analyzed from two perspectives. First, the impact of field-of-view position: in actual antenna surface measurements, target points are primarily located within the central field-of-view. Edge aberrations and nonlinear distortions from the scanning mirror have minimal influence on measurement results. Furthermore, the sensitivity coefficients with respect to the azimuth angle α and elevation angle β vary gradually over small angular ranges. Regarding potential nonlinear effects on the measurement range, analysis of the sensitivity coefficient expressions shows that all S-related sensitivity coefficients are monotonically decreasing functions of 1/S. Within the 1–5 m measurement range, the distance-related sensitivity coefficients vary smoothly without nonlinear distortion. The entire error model and calibration parameters are highly applicable throughout the measurement range.

In summary, this chapter presents a comprehensive analysis of calibration experimental results for the FMCW measurement system. Through zero-point calibration and multi-station measurements, sufficient experimental data were collected. The L-M algorithm was used to estimate the system’s error parameters. Independent verification demonstrated that the calibrated accuracy was significantly improved.

## 4. Discussion

This paper presents a comprehensive calibration method for the FMCW lidar system used to measure the surface of the large-aperture antenna for the Fengyun-4 microwave-sensing satellite program. The research addresses measurement accuracy limitations caused by geometric structural errors and optical axis deviations in complex scanning lidar configurations. The primary contributions of this study include: analyzing and establishing a systematic mathematical error model that incorporates rotational axis tilt error, rotational axis plane misalignment error, scanning mirror deviation, and laser incidence offset. Under the small-error assumption, a first-order linear superposition error propagation model was derived. By combining spatial distance correction with multi-station measurements, the approach eliminates reliance on absolute coordinate accuracy by constructing a point-pair distance residual objective function. The L-M algorithm, integrated with iterative reweighted least squares (IRLS), achieves robust error parameter estimation, while K-fold cross-validation adaptively selects optimal regularization parameters to suppress overfitting. The experiment employed a calibration scheme with four operating conditions and a validation scheme with three independent operating conditions. Through error propagation analysis, the distance measurement uncertainty was determined to be 0.251 mm, and the comprehensive uncertainty for three-dimensional point positions was 17.2 mm. After calibration, the RMS error for relative distances between two points in the validation scenario decreased from 17.5 mm to 2.3 mm. The spatial point cloud ICP registration residuals decreased from a mean of 13.7 mm to 2.5 mm, and point cloud shape retention improved by 86.6%. Simultaneously, validation across three distinct operating conditions confirmed the method’s generalization capability and robustness, providing a reference for high-precision calibration of similar scanning lidar systems.

## Figures and Tables

**Table 1:** Sensitivity coefficient table.

Sensitivity Coefficient	Expression	Sensitivity Coefficient	Expression
$\partial S_{4}/\partial e_{1}$	$\cos\beta +\frac{\alpha_{1}\cdot \cos\beta }{\tan(\frac{90-\beta }{2})}$	$\partial \alpha_{4}/\partial T_{x}$	$\approx \frac{1}{S\cdot \cos\beta }$
$\partial S_{4}/\partial e_{2}$	$2\sin(\frac{90-\beta }{2})+\frac{\alpha_{2}\cdot \cos\beta }{\sin(\frac{90-\beta }{2})}$	$\partial \alpha_{4}/\partial A_{x}$	−cosα
$\partial S_{4}/\partial \alpha_{1}$	$\frac{e_{1}\cdot \cos\beta }{\tan(\frac{90-\beta }{2})}$	$\partial \alpha_{4}/\partial A_{y}$	sinα
$\partial S_{4}/\partial \alpha_{2}$	$\frac{e_{2}\cdot \cos\beta }{\sin(\frac{90-\beta }{2})}$	$\partial \beta_{4}/\partial E_{x}$	≈−cosβ
$\partial \alpha_{4}/\partial \alpha_{1}$	tanβ	$\partial \beta_{4}/\partial E_{y}$	sinβ
$\partial \alpha_{4}/\partial \alpha_{2}$	$\frac{1}{\cos\beta }$	$\partial \beta_{4}/\partial \beta_{3}$	$\approx \frac{L_{0}}{S}$
$\partial \alpha_{4}/\partial \alpha_{3}$	$\frac{L_{0}}{S\cdot \cos\beta }$	$\partial \beta_{4}/\partial T_{y}$	$\approx \frac{1}{S}$

**Table 2:** Relative positions of high-precision reference gauge targets.

Target Point	Coordinate the Position of the Target Point		Relative Distance
P1	−0.09767		
P2	99.9630	P1–P2	99.8653
P3	199.8654	P1–P3	199.7677
P4	299.9220	P1–P4	299.8243
P5	399.9737	P1–P5	399.8760
P6	499.9390	P1–P6	499.8413
P7	599.9704	P1–P7	599.8727
P8	699.9670	P1–P8	699.8693
P9	799.8787	P1–P9	799.7810
P10	899.9317	P1–P10	900.0294
P11	999.9080	P1–P11	1000.0057

**Table 3:** Coordinates of reference target points (unit: mm).

Reference Point	X	Y	Z
P1	−353.916031	581.241957	71.409689
P2	−231.911256	587.59416	96.382430
P3	−86.816273	595.648706	123.231456
P4	−332.861423	605.76438	−50.24143
P5	−209.335451	612.572108	−26.971376
P6	−60.291253	620.684316	1.054062
P7	−307.520477	633.676523	−188.740015
P8	−187.762014	637.220701	−149.484321
P9	−35.811263	649.246139	−141.064106

**Table 4:** Measurement data at Y = 0 mm.

Y = 0 mm				Spatial Angle in the Camera Coordinate System	
Target Point	Measurement Distance	Scene Coordinate X	Scene Coordinate Y	Elevation Angle	Azimuth Angle
P30	4580.353	767.833	470.599	−2.0601	−12.63440
P31	4560.628	765.231	471.359		−10.14666
P32	4544.97	762.435	470.378		−7.63283
P33	4533.98	761.02	469.57		−5.08672
P34	4527.334	760.08	470.373		−2.52343
P35	4525.243	760.733	470.83		0.05562
P36	4527.384	761.214	470.51		2.63125
P37	4534.091	762.954	471.161		5.18285
P38	4545.554	764.821	470.335		7.72484
P39	4561.233	767.33	470.593		10.25859
P40	4581.218	769.243	470.725		12.73534

**Table 5:** Data for position 1.

Target Point	Measurement Distance L	Azimuth Angle α	Elevation Angle β
P1	2533.63	29.7844	−5.7791
P2	2545.32	26.9852	−5.6406
P3	2566.53	23.7046	−5.4157
P4	2523.47	29.8798	−2.5026
P5	2535.14	27.0434	−2.4116
P6	2557.12	23.6612	−2.3090
P7	2519.77	29.8569	1.2605
P8	2531.31	27.0474	0.8111
P9	2553.34	23.6665	1.2925

**Table 6:** Error source uncertainty contribution table (S = 2500 mm, β = 5°, α = 20°).

Source of Error	Contribution to σ_S4_ (mm)	Contribution to σ_α4_ (″)	Contribution to σ_β4_ (″)
$e_{1}$	0.1500	-	-
$e_{2}$	0.2000	-	-
$\alpha_{1}$	0.0083	6.3	-
$\alpha_{2}$	0.0243	144.6	-
$\alpha_{3}$	-	907.0	-
$\beta_{3}$	-	-	300
$T_{x}$	-	8.3	-
$T_{y}$	-	-	8.3
$A_{x}$	-	1015	-
$A_{y}$	-	246	-
$E_{x}$	-	-	7.2
$E_{y}$	-	-	62.8
**Total**	**0.2510**	**1391**	**307**

Note: The above condition parameters are selected based on the typical operating range of antenna array measurements.

## Data Availability

The original contributions presented in this study are included in the article. Further inquiries can be directed to the corresponding author.
